# ECMWF short-term prediction accuracy improvement by deep learning

**DOI:** 10.1038/s41598-022-11936-9

**Published:** 2022-05-12

**Authors:** Jaroslav Frnda, Marek Durica, Jan Rozhon, Maria Vojtekova, Jan Nedoma, Radek Martinek

**Affiliations:** 1 Department of Quantitative Methods and Economic Informatics, Faculty of Operation and Economics of Transport and Communications, University of Zilina, 01026 Zilina, Slovakia; 2 Department of Telecommunications, Faculty of Electrical Engineering and Computer Science, VSB Technical University of Ostrava, 70833 Ostrava-Poruba, Czech Republic; 3 Department of Cybernetics and Biomedical Engineering, Faculty of Electrical Engineering and Computer Science, VSB Technical University of Ostrava, 708 00 Ostrava-Poruba, Czech Republic

**Keywords:** Climate sciences, Environmental sciences, Mathematics and computing

## Abstract

This paper aims to describe and evaluate the proposed calibration model based on a neural network for post-processing of two essential meteorological parameters, namely near-surface air temperature (2 m) and 24 h accumulated precipitation. The main idea behind this work is to improve short-term (up to 3 days) forecasts delivered by a global numerical weather prediction (NWP) model called ECMWF (European Centre for Medium-Range Weather Forecasts). In comparison to the existing local weather models that typically provide weather forecasts for limited geographic areas (e.g., within one country but they are more accurate), ECMWF offers a prediction of the weather phenomena across the world. Another significant benefit of this global NWP model includes the fact, that by using it in several well-known online applications, forecasts are freely available while local models outputs are often paid. Our proposed ECMWF-enhancing model uses a combination of raw ECMWF data and additional input parameters we have identified as useful for ECMWF error estimation and its subsequent correction. The ground truth data used for the training phase of our model consists of real observations from weather stations located in 10 cities across two European countries. The results obtained from cross-validation indicate that our parametric model outperforms the accuracy of a standard ECMWF prediction and gets closer to the forecast precision of the local NWP models.

## Introduction

**Figure 1:**
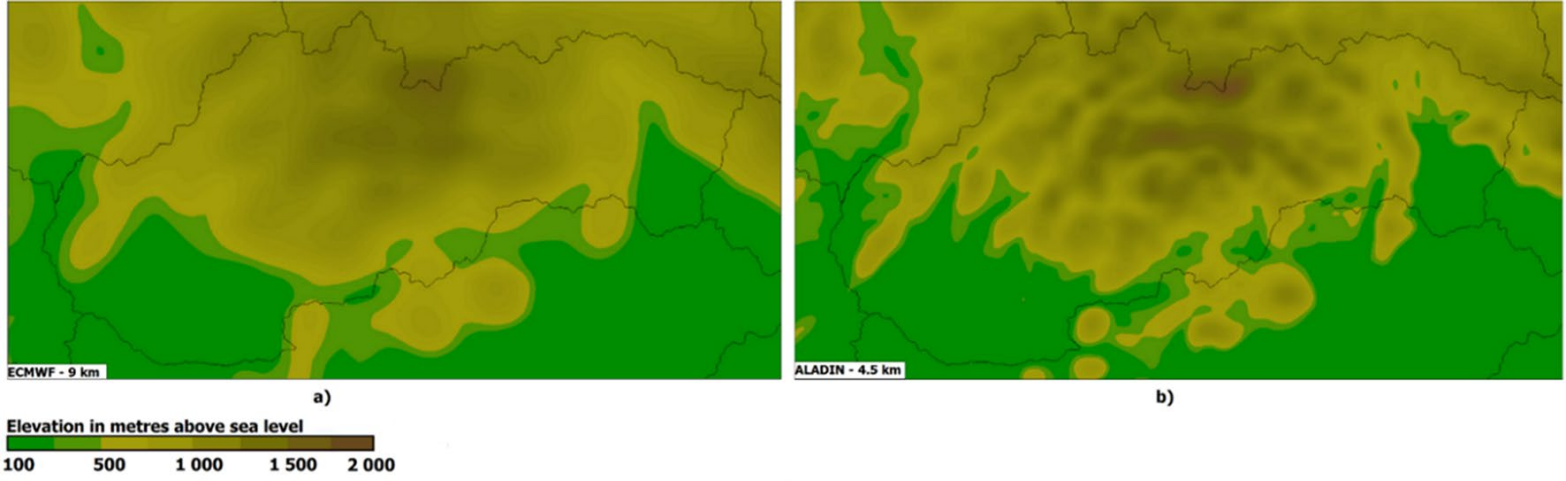
Comparison of NWP horizontal resolution model topography of Slovakia: (**a**) ECMWF; (**b**) ALADIN.

Atmospheric conditions have a significant impact on humans everyday activities. Natural events, such as heavy rains, extreme wind or high temperature, can interrupt air and public transportation services, destroy crops or, even worse, be responsible for fatalities and injuries. To avoid this, the weather forecast is an essential part of many early warning systems aiming at disaster risk reduction. Numerical weather prediction (NWP) models are based on mathematical equations representing the physical behaviour of the atmosphere. The atmosphere can be described in terms of its properties such as temperature, humidity, or precipitation rate. Atmospheric modelling uses physical parameterization, which means that many complex processes in the atmosphere, such as radiative and cloud processes or atmospheric turbulence, are represented in the model by simple and computationally inexpensive methods. Evaporation, vegetation cover, or reflection/absorption of solar radiation at the Earths surface represent examples of processes that are often parametrized (the main purpose is modelling the effects of these processes). These processes affect too a small area to be predicted in full detail by NWP models. The major reason lies in the limited computing power that still does not allow us to calculate the above-mentioned processes for any place on Earth. As a result of this, the climate model (mathematical simulation of the Earth’s climate system) is divided into a three-dimensional set of points a grid characterizing the horizontal and vertical resolution of the NWP model. Grid-spacing has an impact on complex spatial modelling of surface terrain (see Fig. 1), as well as on representation of the atmosphere (number of layers across the height of the atmosphere). Based on the grid spacing and forecast period, NWP models can be divided as follows:

- *Global NWP models* resolution of 9 km (ECMWF) or of 13 km like Global Forecast System (run by the United States National Weather Service,), more than 10 days of prediction.
- *Local NWP models* less than 5 km resolution, the forecast horizon is 3 days. Some of the well-known European models comprise ALADIN (operated in 16 countries) or HIRLAM (operated in 10 countries). As for local NWP models, the data from global models supply the data for the lateral boundary conditions.

The greater the distance between the grid points, the less likely the model will be capable of discovering small-scale variations in the temperature and moisture fields. The lack of resolution reduces the amount of detail and increases the prediction error.

As for local (regional) NWP models, it is characteristic that each country (represented by the national weather service) calculates the weather forecast individually according to its own computational capacity and needs (e.g., some countries can use the ALADIN horizontal resolution 11 km, others have applied the improved version of resolution of 4.5 km)^1^. We have identified two major disadvantages of this approach:

- Ineffective searching of weather information from the user’s perspective. There is no common website collecting data from local NWP models. Not all countries make this data open access, so the potential users must pay for it.
- The national weather agencies must invest a large amount of money to keep their high computing infrastructure up to date.

On the other hand, several popular web applications collect and visualize data from global models, therefore, they allow users to find information about the weather conditions for any place. Consequently, we have decided to calibrate the ECMWF global model by a neural network (hereinafter referred to as NN) to bring 3-day weather forecast accuracy as close as possible to the selected reference local model ALADIN. Because the 72-hour forecast period is a standard setting for the ALADIN model, our main objective is to improve ECMWF forecasts to this time window.

Horizontal resolution can be considered as a major weakness of the ECMWF model. The grid spacing of 9 km means that a geographic area of 81 square kilometres is approximated only by one grid point approaching the average of the observed values within the grid cell. The region of Central and East Europe consists of several small countries with a population of less or around 10 million (Slovakia, Czechia, Austria, Slovenia, Hungary, or Croatia). Due to this fact, the whole area of small and mid-sized cities dominating the settlement structure of these countries is geographically approximated by one or two topographic points of the ECMWF grid. As a result of that, the model poorly approximates the terrain and local micro-climate parameters we have recognised as important weather features, such as green infrastructure or local pollution. These features either exist in the model initial conditions, but are weakly represented, or are barely incorporated into the model initial conditions. Both situations will impact the forecast. Therefore, after the comprehensive study of the works published, as well as thanks to the knowledge received from the previous work^2^, we have identified key features that should effectively replace the main advantage of local NWPs higher resolution. Appropriate features selection helped us adequately incorporate the influence of small-scale phenomena into the ECMWF forecast, especially in urban areas.

The main contributions of this paper include the following:

- We describe our approach on improving the accuracy of the ECMWF model based on machine learning (ML). ML is used for post-processing raw ECMWF data.
- We present a novel perspective of selecting small-scale phenomena that are able to correct a 2 m air temperature and a 24 h accumulated precipitation forecast error.
- The proposed model can be easily incorporated (open source) into potential online forecast web services; due to simple model topology, it is robust to overfitting.

The rest of the paper is organised as follows: Section State of the Art reviews some important works in this research topic. In section Methodology, the details of our hybrid NWP-ML model are presented. Section Results describes model performance analyses and comparison with the reference local model ALADIN. The benefits and limitations of the proposed approach are discussed in section Discussion. Summary and future work plans are presented in section Conclusion.

## State of the art

Modern weather prediction systems strongly rely on massive mathematical modelling and simulations which require high-performance computing. Although we witness a fast increase in computing power, there are still various challenges in NWP. Regional NWP models need to have accurate information for all forecast variables and along each model boundary (secured by selected global models), but, due to the unpredictable events in the atmosphere, even a small change in the initial stage can lead to a huge impact on weather forecast^3^. Lower resolution of global NWP models can take into account even micro-climates such as soil moisture or vegetation cover, but in a very limited manner. Our suggestion that these parameters are relevant for improving the accuracy of the forecasts has also been confirmed in the study by Srivastava and Blond^4^ and Dirmeyer, P. A., and Halder, S.^5^, where the authors proved that the aerosols distribution/concentration and accurate initialization of soil moisture are both important indicators that can improve air temperature and rainfalls forecasts delivered by global NWP models. Jhun et al.^6^, Li et al.^7^, Perrone et al.^8^, Requia et al.^9^, Manso et al.^10^, Tomson et al.^11^, Zhao et al.^12^, and Schwaab et al.^13^ have investigated the influence of air pollution as well as the green infrastructure on weather changes too. Seasonal temperature or precipitation frequency correlates especially on the level of particulate matter (especially PM2.5). An investigation of the so-called urban heat islands effect brought important information on how to improve thermal comfort in urban areas. Green, or sometimes called blue-green, infrastructure (parks, green walls, wetlands, city forests) help capture pollution and mitigate climate change (cooling effect, water retention).

The deep neural network is a kind of NN consisting of several layers called hidden layers interconnecting the vector of input features with the output layer. More layers allow resolving more complex fitting issues and provide good generalization over the selected dataset. A set of input parameters (features) has to be chosen carefully; individual parameters should not be cross-correlated. If the NN topology contains many layers and neurons on each layer, there is a potential risk of overfitting and additional methods for overfitting reduction must be applied. In the last years, research teams have been increasingly aware of deep learning and trying to use a benefit of it to improve numerical modelling or apply it for additional post-processing of the delivered NWP forecasts. In studies by Rasp and Lerch^14^, Yonekura et al.^15^, Wang et al.^16^, Ren et al.^17^, and Schultz et al.^18^, the authors tested the possibility of replacing the traditional forecast model completely by NN or using it for post-processing of the delivered forecast. While some promising results, that can compete with NWP, have been identified in pilot models, the research is still in the beginning. A much better situation is in the area of a hybrid approach where the NWP data are post-processed by NN. Many of the works mentioned focused their attention on the improvement of nowcasting (up to several hours ahead) or a one-day lead. As for heavy rainfalls forecast, in particular, the authors reached significant accuracy improvement.

We have also contributed to this research. In our pilot work^2^, we proposed the initial version of our hybrid model for ECMWF accuracy improvement. We verified that our idea is correct and NN can help improve prediction accuracy. Now, the weather data collected contain three times more samples than we previously used for the training. We also increased the model’s minimum and maximum forecast range for both the hourly temperature and the daily precipitations. With respect to the contributions of the above-mentioned works and our knowledge gained, we changed the NN architecture and replaced two input features. The results obtained are found to be superior compared to our previous work; and to our best, we have not discovered any model published that offers ECMWF model accuracy improvement for the 3-day forecasts for both the 2 m air temperature and the daily precipitation concurrently.

## Methodology

**Figure 2:**
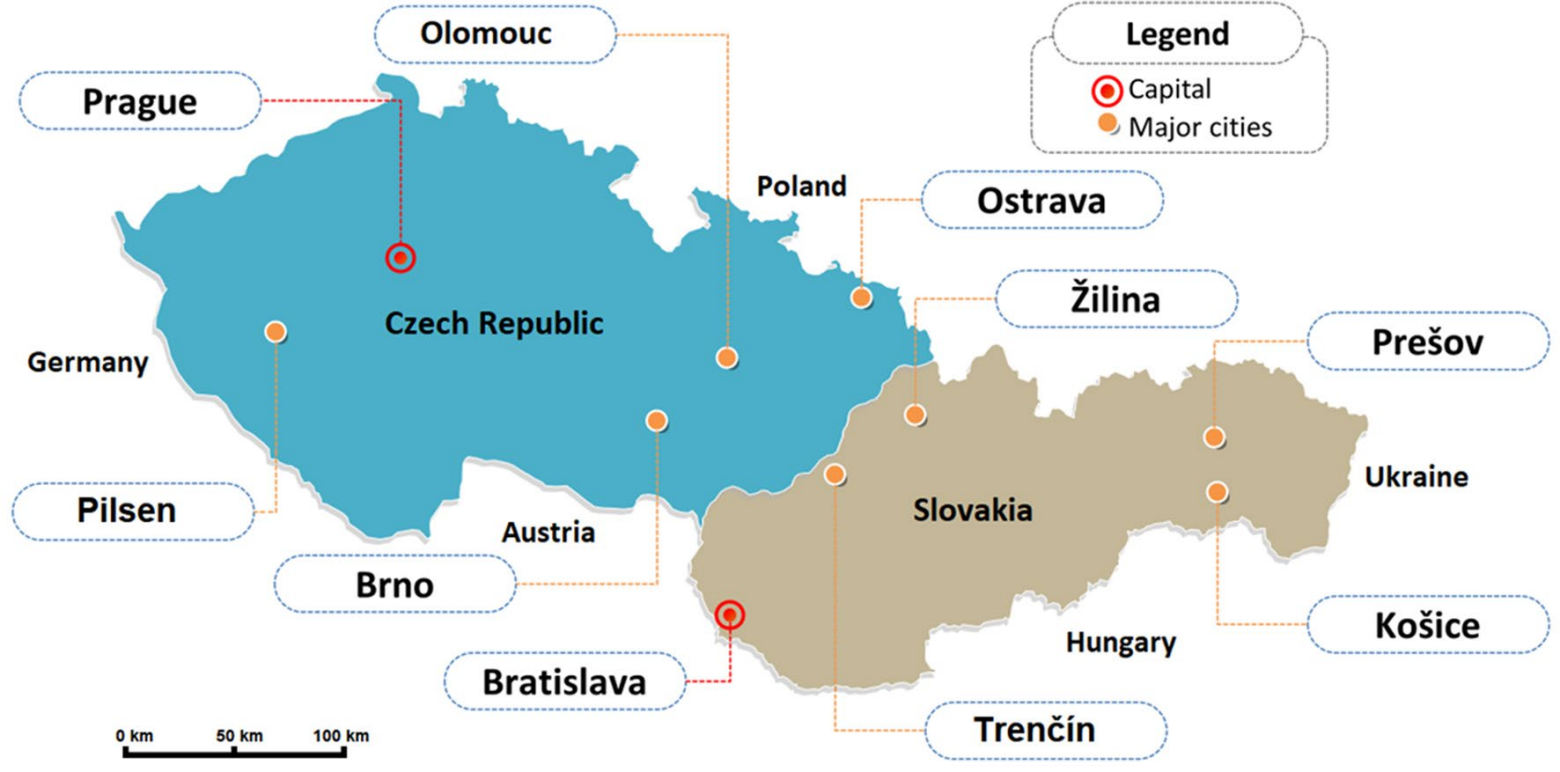
The geographical location of selected cities.

At first, we needed to create a large database containing the predicted values of the NWP models selected and the values measured by weather stations (ground truth). More than one year was spent on collecting the data. We interrupted gathering the data by the end of February 2020 due to the new coronavirus COVID-19 spreading across the world. The lockdowns imposed in many countries caused the weather forecasts to be less accurate due to reduction in aircraft weather reports^19^. Had we not interrupted the data collection, we would have received noisy data. For the short-term forecast, we used open-access data from several web services, namely windy.com (which uses raw ECMWF data with a 3-hourly step that allows producing forecasts up to 144 h ahead), Yr.no (operated by Norwegian Meteorological Institute MET; raw ECMWF data with a 1-hour step frequency that allows a forecast of approximately 60 h ahead; for a longer period forecast, the step increases to 6 h which can influence total precipitation forecast accuracy for 3rd day) and, finally, the Czech and Slovak Hydrometeorological Institutes (both use the ALADIN regional model that is run on their supercomputers)^20–23^. Weather services in Czechia and Slovakia also served as sources of real measured values obtained from surface weather observation stations. All weather stations selected are located near the centre of the following cities (see Table 1 and Fig. 2)

**Table 1:** List of selected cities.

**City**	**Population**	**Area [km**$^2$**]**
Prague the capital city of Czechia	1.335 million	496
Brno	382,400	230
Ostrava	285,000	214
Olomouc	100,500	103
Pilsen	175,200	138
Bratislava: Capital city of Slovakia	475,600	368
Zilina	81,900	80
Kosice	227,500	243
Presov	83,900	70
Trencin	54,500	82

The whole database is composed of 3,679 unique samples. The database was randomly divided into a set dedicated to the training procedure (90% of samples) and a cross-validation set (10% 368 samples). The database reflects the limitations of the models, such as 3-hour resolution (ECMWF) and maximal 72-hour lead time (ALADIN). We collected forecasts from all NWP models delivered by forecast run at 00:00 UTC (Coordinated Universal Time). In the dataset, each date of forecast run (denoted as d) of a particular city is associated with its Air Quality Index (AQI) of the previous day (d-1) from the website (AQI^24^).

PM2.5 AQI is calculated according to the PM2.5 level in the air. These matters of size of less than 2.5 $\mu$m are recognized as a major air pollutant with an impact on weather conditions as is stated in section State of the Art.

Green infrastructure has been incorporated in our model via two components, namely the urban water area surface and the Mean Effective Green Infrastructure (MEGI)^25^ . The MEGI indicator shows the spatial distribution of Effective GI, which can be explained as the probability of finding a GI element in a selected urban area. It is based on circular regions (the distance between two circles is 1 km) around the city centre. Since we collected data from weather stations situated close to the city centres, each city in our database is associated with the MEGI value reflecting a 5 km distance to the city centre. The list of all dataset variables is shown in Table 2.

**Table 2:** List of dataset variables.

**Parameter type**	**Description**
Climatic variables	Hourly surface air temperature, daily precipitation
Weather forecast models	ECMWF, ALADIN, Yr.no
Target data	Meteorological data from weather stations
Day of forecast	1st, 2nd or 3rd day
Microclimate attributes	MEGI, AQI, water area surface

### Architecture of hybrid NWP/ML prediction model

**Figure 3:**
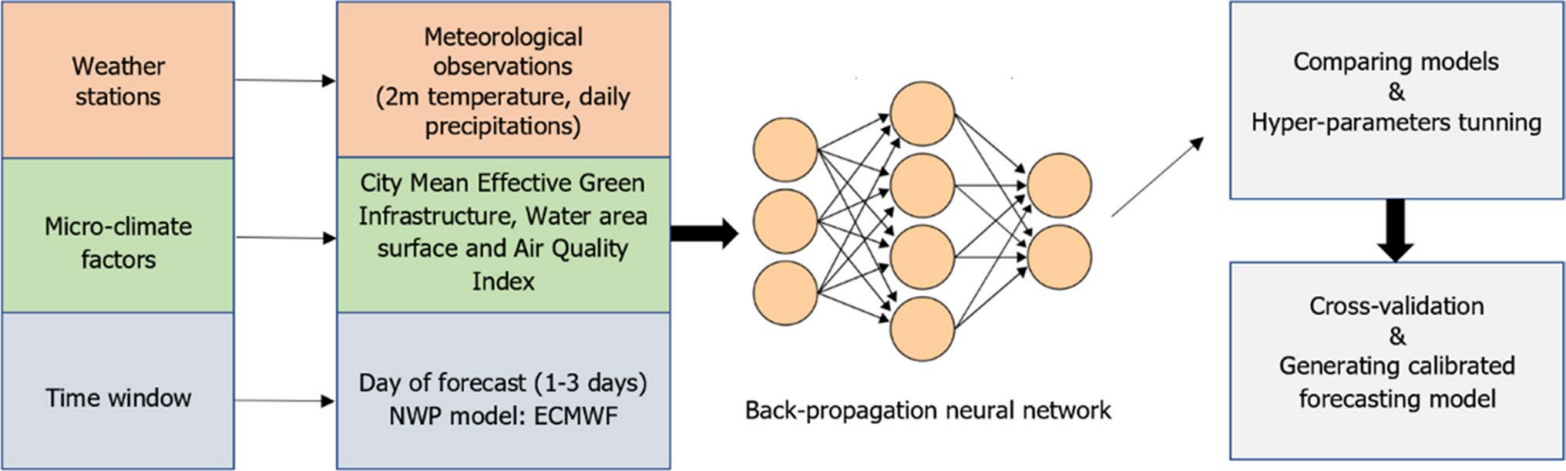
The architecture of the proposed model.

A neural network based on error back-propagation comprised an input layer, a hidden layer (one or more) and, finally, an output layer. This topology is inspired by the learning process of the human brain (creation of new neural connections leads to storing new information). The input layer is represented by one or more elements of the input vector called features. The whole process of NN training and testing is shown in Fig. 3.

Back-propagation neural network is not the only type of neural network. Other well-known types are the Long Short-Term Memory (LSTM) network or the Convolution Neural Network (CNN). LSTM works with sequence data such as time series or speech recognition data because the network can store past important information due to its recurrent connection on the hidden state. Another network CNN applies filters (kernels) that, by using the convolution operation, allow extracting spatial features e.g., from an image. Therefore, CNN is good for classification. Because we have tabular data (and create regression model) and, we do not use radar images or try to predict weather from its previous state, a back-propagation neural network seems to be the best solution. An example of the input vector *x* of n-th data sample can be expressed as follows:

$$\begin{aligned} x_n = \begin{bmatrix} {Day}\,{of}\,{forecast} \\ AQI \\ MEGI \\ {Water}\,{area} \\ {ECMWF}\,{forecast} \end{bmatrix} = \begin{bmatrix} 1 \\ 66 \\ 15.22 \\ 1.8 \\ 14 \end{bmatrix} \end{aligned}$$

We tested many topologies, starting from simple networks containing one hidden layer to more sophisticated topologies consisting of 3 hidden layers, wherein each of them had tens of neurons. To prevent network overfitting, we applied the following techniques:

- Bias Variance Trade-Off: a compromise between the model size and accuracy. A simpler model topology is preferred.
- Early stopping: training will stop when the preferred performance indicator stops improving on the validation data subset.
- Bayesian regularization: this method minimizes a combination of squared errors and weights, and then decides the best combination to create NN that generalizes well.

A model error is considered as a Mean squared error (MSE) defined as follows:

$$\begin{aligned} MSE = \frac{1}{n} \sum _{i=1}^n \left( y_i - {\widetilde{y}}_i \right) ^2, \end{aligned}$$

where *n* denotes the number of samples and MSE represents the difference between target value $y_i$ and output value (obtained from a trained model) ${\widetilde{y}}_i$ i. The root of MSE (RMSE) is a common metric that shows the standard deviation of residuals (prediction errors) for a particular variable.

A prediction model was implemented in the MATLAB workspace (version 2021a) by using Neural Network Toolbox which allowed to create testing scripts as well as the so-called MATLAB function of the final version for further deployment. As an activation function, we used a predefined hyperbolic tangent sigmoid transfer function (tan-sig). The activation function of each neuron indicates the activation potential of the neuron that is responsible for the decision whether the node will be fired or not. Tan-sig is an S-shaped curve function that produces outputs in a scale of [− 1,1]. The non-linearity of this function helps the model to generalize or adjust better to data diversity.

## Results

After the collection of all data, we performed a basic statistical investigation. At first, we analyzed the relative accuracy of each model prediction outputs. Relative accuracy RA (expressed as a percentage) specifies how accurate a prediction is when compared to the target value. The formula is expressed as follows:

$$\begin{aligned} RA = \frac{\left[ v_t - \left( v_t - v_p \right) \right] }{v_t} \times 100 \end{aligned}$$

where $v_t$ denotes the target value and $v_p$ represents the predicted value.

Table 3 shows that, in terms of relative accuracy, ECMWF has achieved the worst results of temperature forecasts, while ALADIN and Yr.no performed equally well (for both models, the RMSE oscillates between 1.25 and 1.5 for the 2 m air temperature). The calculated RA dedicated to the ECMWF 3-day projection is close to the declared accuracy promoted by ECMWF itself (for year 2019 98%) (ECMWF^26^).

An interesting situation occurred when we compared the daily precipitations forecast accuracy, where the ECMWF model surpassed its two competitors. A detailed analysis related to whether the models overvalue or undervalue their predictions is another curious fact that we have extracted from the dataset. As it can be seen in Table 4, in comparison with the other two models, the ECMWF model typically undervalued the temperature prediction and slightly overvalued the number of daily precipitations.

**Table 3:** Overview of relative accuracies (RA) of weather prediction models produced for both countries.

**Weather model**	**Surface temperature %**	**Daily precipitation %**
ALADIN (SHMU and CHMI)	99.7	89.5
Yr (MET)	99.1	86.9
ECMWF (Windy)	97.6	90.8

We can verify this assumption from another point of view. The total number of real measured hourly temperatures above 30 $^\circ$C is 224. While ALADIN and Yr.no models predicted this warm temperature more than 200 times (concretely ALADIN: 210 and Yr.no: 208), ECMWF estimated that this extreme value would be reached only 183 times. A relatively high portion of correct predictions of total daily precipitation (more than 60%) can be explained by a huge number of zero precipitation forecasts that were truly predicted.

**Table 4:** Comparison of ratios that determine whether the models’ predictions are overvalued or undervalued.

**Weather model**	**Surface temperature**			**Daily precipitation**		
	**−**	**=**	**+**	**−**	**=**	** +**
ALADIN (SHMU and CHMI)	42.1%	18.2%	39.7%	15.6%	61.5%	22.9%
Yr (MET)	40.5%	18.3%	41.2%	16.3%	61.2%	22.5%
ECMWF (Windy)	48.3%	18.3%	33.4%	15.7%	60.1%	24.2%

Note: sign + represents overvaluing,- undervaluing, and = correct prediction.

### Neural network modelling

During the modelling phase, MATLAB allows us to randomly divide the training set into three subsets, namely a training subset, a validation subset and a testing subset (we chose the following ratio: 80-10-10). We have applied early stopping which stops the training procedure when the validation error begins to rise but the training error still decreases in the followings iterations. This event indicates that the network starts to overfit the data. The trained network is then evaluated on testing data. In case the trained model performance is adequate for all three subsets, we can use a cross-validation dataset (sometimes referred to as a holdout) that contains samples not participating in model training.

**Figure 4:**
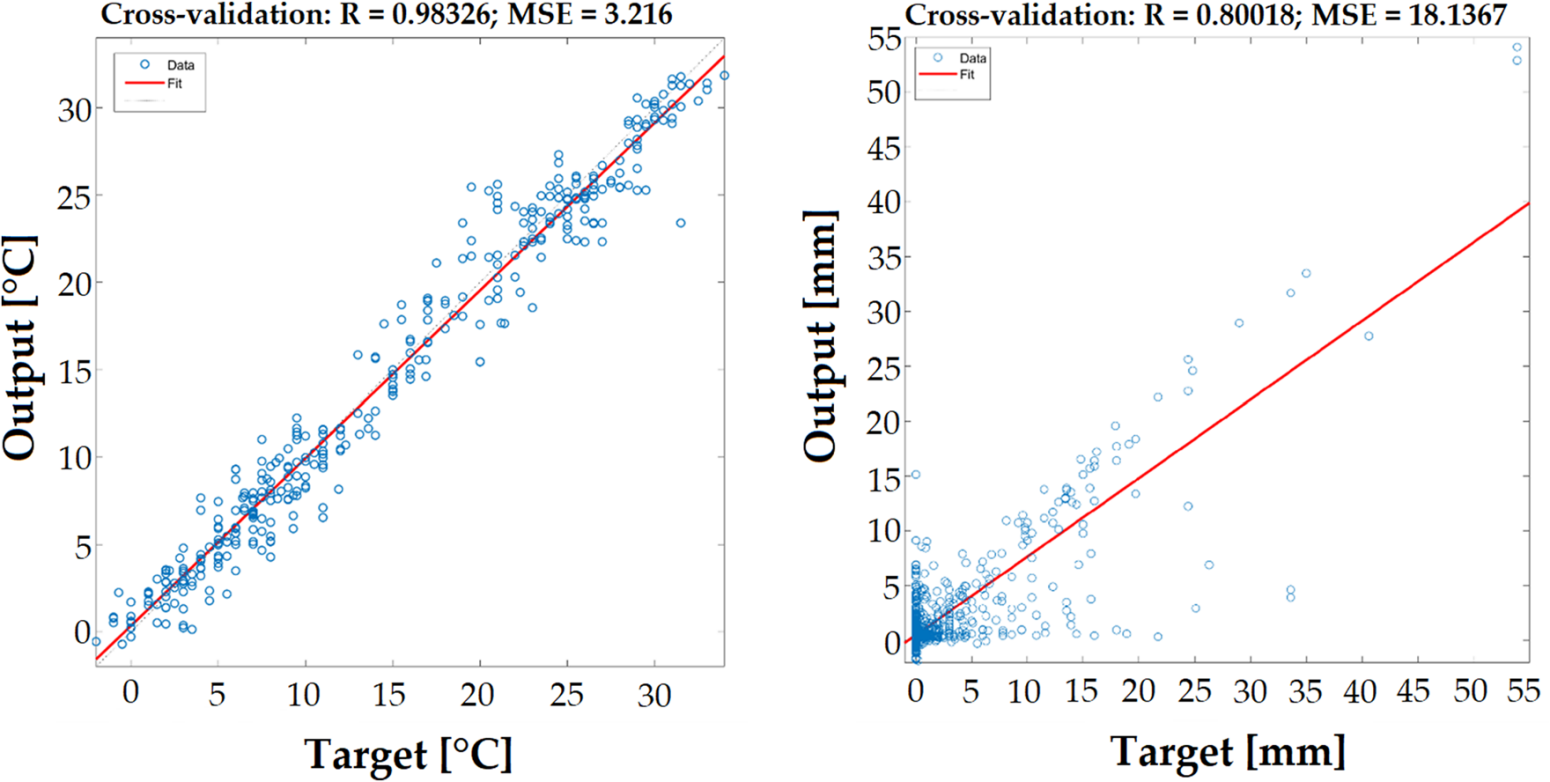
Scatter plot with the Pearsons correlation coefficient R and MSE of hourly temperature prediction in the left part and daily precipitation prediction in the right part.

**Figure 5:**
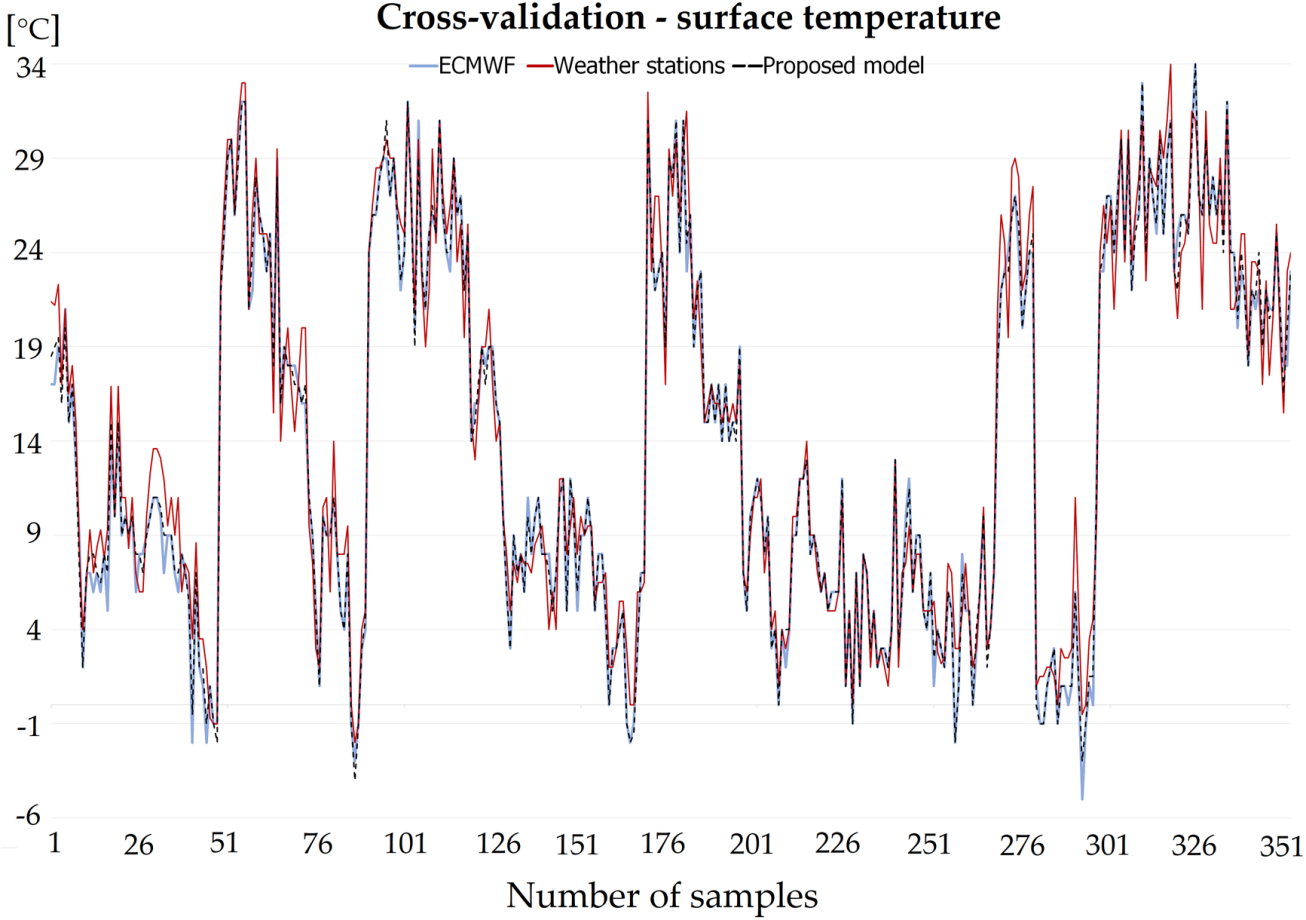
Comparison of predicted surface temperature from the proposed model and ECMWF, and reference temperature from weather stations.

Figure 4 shows a correlation diagram with the Pearson’s coefficient R for the best-rated topologies, concretely a hidden layers topology of 7-5-2 for the hourly temperature prediction and an 11-4 for the prediction of total daily precipitations. Figure 5 compares the proposed model temperature predictions with the raw ECMWF predictions and with the data observed from weather stations.

Based on the results shown in Table 5, we can say that our proposed hybrid model outperformed raw ECMWF predictions. For the cross-validation set, by using the RMSE criterion, we have improved the forecast accuracy by 13% (hourly temperature) and by 45% (daily precipitations).

**Table 5:** Proposed model performance evaluation.

**Weather model**	**Surface temperature**		**Daily precipitation**	
	**MSE**	**RMSE**	**MSE**	**RMSE**
ECMWF	4.44	2.11 °C	42.28	6.5 mm
Proposed model	3.73	1.93 °C	11.49	3.39 mm
**Cross-validation set**				
ECMWF	4.09	2.02 °C	38.17	6.18 mm
Proposed model	3.22	1.79 °C	18.14	4.26 mm

### Comparing the performance of different machine learning algorithms

**Figure 6:**
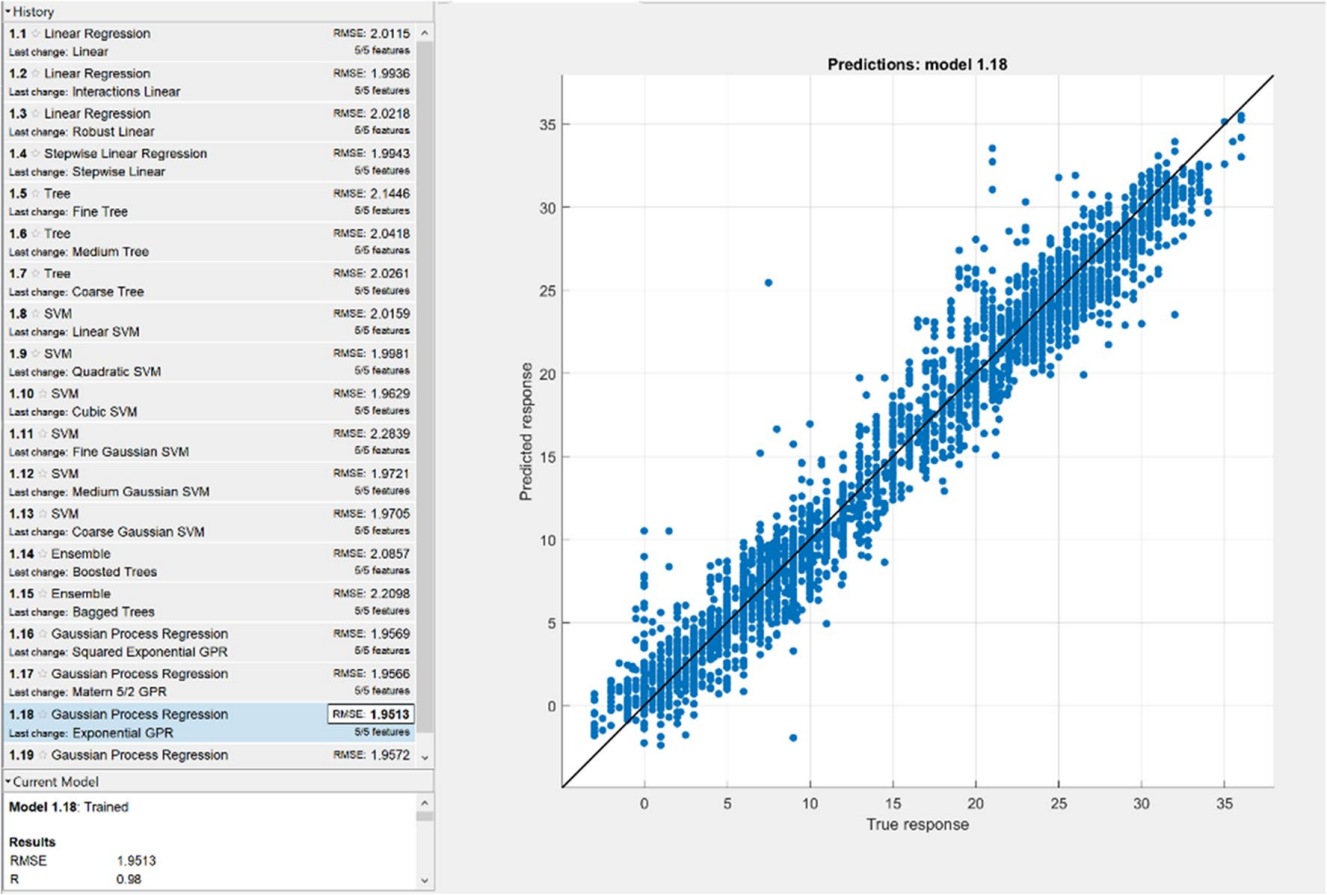
Correlation diagram of selected ML models (RMSE and Pearsons correlation coefficient R) for hourly temperature prediction.

**Figure 7:**
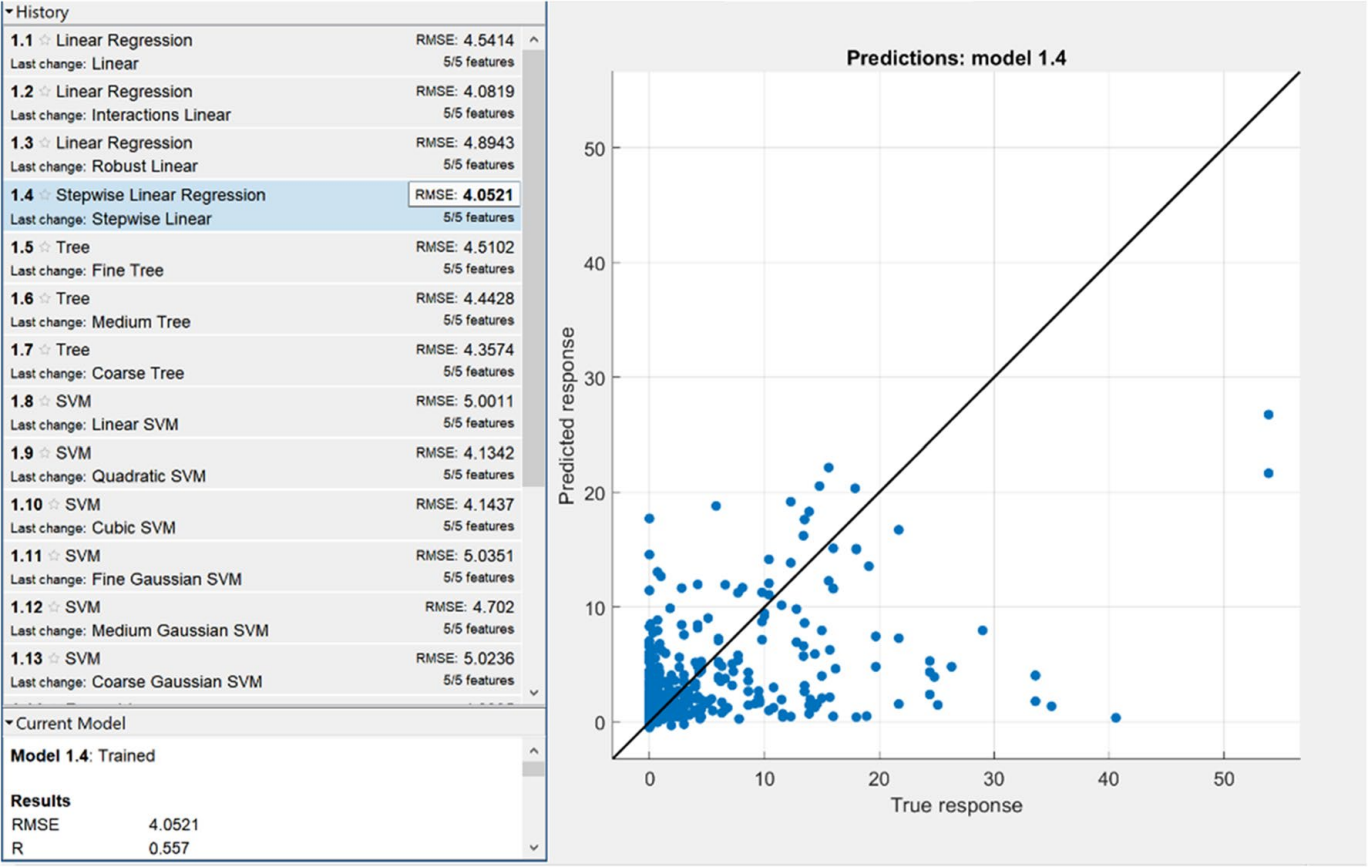
Correlation diagram of selected ML models (RMSE and Pearsons correlation coefficient R) of daily precipitation prediction.

Although NN has been a trendy ML algorithm in recent days, evaluation of other ML techniques is important to come out with the best-suited algorithm for a particular issue. MATLAB contains Statistics, and Machine Learning Toolbox allows us to compare various ML algorithms and build predictive models by automatic training of different regression models on our data. All available data were used for the training. We set 5-fold cross-validation (good compromise that saves time and computational complexity (Marcot and Hanea^27^) to check the models accuracy. Figures 6 and 7 show the results obtained by selected ML algorithms.

As it can be seen in the above-stated figures, performance indicators related to the surface temperature reach the best values in case of using the Gaussian Process Regression (GPR) model (non-parametric Bayesian approach). For the total precipitation forecast, Stepwise linear regression becomes the best-assessed model. Based on these performance measurements, we can say that our proposed ML model outperformed the GPR model (in a form of R as well as RMSE for both cases training and cross-validation). In total daily precipitation prediction, our model achieved better results on the training data (and significantly better R value in cross-validation). The RMSE metric in cross-validation where we received slightly worse outcome (4.26 vs 4.05, 5% difference between the models performance) was the only exception. On the other hand, it needs to be considered that these ML techniques used all available data; before NN training, the dataset was divided into training and cross-validation (independent) data subsets.

Therefore, we have enough information to assess our model truly. Based on these measurements, we can say that the back-propagation neural network selected is the best fit model (for non-linear mapping) with the highest prediction accuracy.

## Discussion

We have prepared a hybrid deep learning model able to correct ECMWF short-time forecasts (up to 3 days ahead). The proposed model significantly extends the pilot study published in 2019 (an increase of forecast ranges from 15 °C − 35 °C to −5 °C − 35 °C; and 24 h precipitation forecast modelling starts from 0 mm instead of 2 mm). The key performance indicator RMSE is also better compared to the pilot study (2.06 C and 3.68 mm the training set of the pilot model). We also acquired three times more unique data samples available for training and testing. As shown in Table 5, the proposed model improved the prediction ability of the raw ECMWF model. The proposed model is comprised of simple NN architecture which implies good generalizing ability and robustness to overfitting. The model is easy to integrate as a post-processing approach for ECMWF outputs. Table 5 also shows that the results obtained for the model during the training and cross-validation are generally consistent and MSE falls close to the values represented by the local NWP model. A combination of deep learning and global NWP outcome can offer similar prediction accuracy as the local model.

We should consider and discuss the limitations of this study. Zero precipitation rate accounts for more than 66% of all data. There is a noticeable imbalance in the data, and all papers mentioned here had to find methods to effectively handle imbalanced datasets. An imbalanced dataset may have a negative impact on the performance of machine learning, especially as for classification models. Thus, our primary goal was to adapt the model itself by hyper-parameter tuning and monitoring feedback from metrics. We also adopted a strategy described by Liu et al.^28^ to deal with this issue. The precipitation training dataset was a combination of randomly chosen data from a non-rainfall subset as well as from a rainfall data subset. The proportion of occurrence of data extracted from these two subsets is 1:1. This special training dataset was used for the training NN (we used only 1,129 data samples in this case).

Another fact that we must consider is that the daily precipitation summary is valid for a period from midnight to midnight. A situation may occur when rain is expected e.g., at 22:00, but the rainfall starts after midnight. Only a few hours delay will affect the predictive accuracy for the actual and following days of the forecast. Finally, the temperature prediction range represents a drawback of the proposed model. Due to the relatively warm winter, we have recorded hourly temperatures below minus 5 degrees of Celsius only a few times. There is no doubt that, in many countries, the lowest temperature during winter is much lower than the actual model offers. Unfortunately, we must wait until the situation with air traffic returns to its pre-COVID levels, and, then, we can focus on gathering data with low surface temperature to improve the prediction range of our model.

It is not easy to find research papers for comparison. Many authors have turned their attention to improvement of the prediction accuracy for nowcasting (up to next 12 h) or extreme weather phenomena such as rainfalls or monsoons. Nevertheless, we have found several interesting articles. Li et al^29^ tested linear and Random Forest algorithms to improve the ECMWF forecast in the Beijing area. They gathered data during the period between 2012 and 2016. The proposed model increases accuracy by 9.61% compared to the ECMWF for surface temperature prediction. Another group of Chinese researchers in Kong et al.^30^ created a model for a weather conditions forecast for the upcoming 3 days. They gathered historical observational data from 226 weather stations across Beijing between 2015 and 2017. The proposed model was based on the convolutional neural network containing 44 forecast predictors. While the ECMWF model reached RMSE of 2.94, their trained model reduced the error to 2.41 in temperature forecasting. In Thi Kieu Tran et al.^31^, the authors tested three machine learning algorithms. In addition to NN, they also used the recurrent neural network (RNN) and LSTM. According to the season of the year, RMSE oscillated between 2.52 and 3.65 $^\circ$C in the data test of the daily maximum temperature prediction for the upcoming 3 days. Error comparison with the data observed is missing.

In ECMWF technical memorandum number 896^32^, authors analyzed three machine learning algorithms (linear regression, random forests, and NN) for statistical modelling of 2 m temperature and 10 m wind speed forecast errors. Compared to our model, their corrected model produced a forecast with a lead time up to only 48 h and their models’ configurations often used more than 10 predictors (features). RMSE was reduced by around 10%. NN model outputs were slightly better than the other two mentioned methods, its RMSE oscillates between 1.9 and 2.4 $^\circ$ for 48 h forecast lead time.

Better short-term precipitation prediction from radar echo images was the main contribution of the paper by Niu et al.^33^, where the authors used a combination of multi-channel ConvLSTM and 3D-CNN algorithms. Based on the data collected between 2017 and 2018, their model achieved an RMSE value of 7.18 mm. The preprint paper by Chen and Wang^34^ describes an application of DL in a short-term precipitation prediction (following 24 h). The proposed model reached smaller RMSE (4.16 mm/24 h) than the ERA-Interim 24 h forecast (4.78 mm/24 h) that estimates the global atmospheric state based on the data from ECMWF. Saminathan et al.^35^ calibrated the delivered precipitation forecast provided by Global Ensemble Forecast System (GEFS) over the Indian region. They used NN as a post-processing technique for forecast enhancement. The prediction error represented by RMSE was improved from 9.8 to 9.1 for a 3-day lead. Finally, Rasel et al.^36^ tested ML models based on Support Vector Regression (SVR) and NN for weather forecast accuracy improvement for a time period of one week. In terms of RMSE, the best models with a low error rate included SVR (with its value of 21.68 mm/24 h) and NN (with an error rate of 7.89 $^\circ$C).

Based on the above-mentioned papers published, it is obvious that the combination of temperature and precipitation forecast accuracy improvement is unique in this research field. In addition, our paper brings a calibration model that can be easily incorporated into popular online weather services.

## Conclusions

This paper proposes ECMWF model calibration based on DL. The novelty of this approach lies in prediction accuracy improvement for both 2 m air temperature and daily precipitation in a 3-day lead. Performance analysis of our model pointed out an error (RMSE) reduction by 13% (2 m air temperature) and 45% (24 h precipitation) respectively. In comparison to regional NWP models, the global model ECMWF has no geographical restrictions in forecasts provided, thus it is more attractive for both professionals and basic users. It is also worth mentioning that our approach could potentially reduce the acquisition costs of supercomputers. For example, in 2018, the Czech hydrometeorological institute bought a supercomputer with a theoretical peak performance of 270 Teraflops (NEC LX-Series x86 with 320 nodes). On the other hand, we tested our model on PC Intel(R) Core(TM) i7-9700 CPU @ 3.00 GHz with 16 GB RAM, and the corrected ECMWF predictions were calculated in the MATLAB environment almost immediately (CHMI^21^). In future work, we plan to extend the calibrated forecast period and incorporate another weather phenomenon the wind speed.

## Data Availability

The data sets source code of proposed models are available online in Zenodo repository (https://doi.org/10.5281/zenodo.5862250). The air quality and meteorological data used in this work are public and freely available from ECMWF, Yr.no, SHMU, CHMI, and AQI.
